# Generation and characterization of genetically and antigenically diverse infectious clones of dengue virus serotypes 1–4

**DOI:** 10.1080/22221751.2021.2021808

**Published:** 2022-01-07

**Authors:** Tomokazu Tamura, Jiayu Zhang, Vrinda Madan, Abhishek Biswas, Michael P. Schwoerer, Thomas R. Cafiero, Brigitte L. Heller, Wei Wang, Alexander Ploss

**Affiliations:** a Department of Molecular Biology, Princeton University, Princeton, NJ, USA; b Research Computing, Office of Information Technology, Princeton University, Princeton, NJ, USA; c Carl Icahn Laboratory, Lewis-Sigler Institute for Integrative Genomics, Princeton University, Princeton, NJ, USA

**Keywords:** Dengue, dengue virus, host tropism, animal models, host response

## Abstract

Dengue is caused by four genetically distinct viral serotypes, dengue virus (DENV) 1-4. Following transmission by *Aedes* mosquitoes, DENV can cause a broad spectrum of clinically apparent disease ranging from febrile illness to dengue hemorrhagic fever and dengue shock syndrome. Progress in the understanding of different dengue serotypes and their impacts on specific host-virus interactions has been hampered by the scarcity of tools that adequately reflect their antigenic and genetic diversity. To bridge this gap, we created and characterized infectious clones of DENV1–4 originating from South America, Africa, and Southeast Asia. Analysis of whole viral genome sequences of five DENV isolates from each of the four serotypes confirmed their broad genetic and antigenic diversity. Using a modified circular polymerase extension reaction (CPER), we generated *de novo* viruses from these isolates. The resultant clones replicated robustly in human and insect cells at levels similar to those of the parental strains. To investigate *in vivo* properties of these genetically diverse isolates, representative viruses from each DENV serotype were administered to NOD Rag1^−/−^, IL2rg^null^ Flk2^−/−^ (NRGF) mice, engrafted with components of a human immune system. All DENV strains tested resulted in viremia in humanized mice and induced cellular and IgM immune responses. Collectively, we describe here a workflow for rapidly generating *de novo* infectious clones of DENV – and conceivably other RNA viruses. The infectious clones described here are a valuable resource for reverse genetic studies and for characterizing host responses to DENV *in vitro* and *in vivo*.

## Introduction

Dengue Virus (DENV) is a mosquito-borne viral pathogen primarily transmitted by two species of mosquito: *Aedes aegypti* and *Aedes albopictus*[1]. Since the 1940s, the number of people infected with DENV has been steadily increasing due to globalization of travel[2], rapid urbanization[3], and a scarcity of vector control programs[4]. Epidemiological models estimate a total of 390 million annual dengue infections, leading to 96 million symptomatic infections and approximately 10,000 deaths each year[5,6]. Currently, dengue is endemic to at least 128 tropical and subtropical countries, placing almost 3.9 billion people at risk of infection[6]. Over the past half century alone, the global prevalence of dengue has increased 30-fold, with a 500-fold increase in the most severe clinical manifestations such as dengue hemorrhagic fever and dengue shock syndrome[7,8]. With the geographic distribution of disease continuing to expand, dengue remains the leading cause of arthropod-borne viral disease in the world[6].

Dengue viruses are classified as enveloped, positive sense single stranded RNA viruses of the *Flaviviridae* family, which can be subdivided into four distinct serotypes: DENV-1 through DENV-4[9]. The four DENV serotypes differ from one another by 25–40% at the amino acid level and can be further classified into separate genotypes that vary up to 3%[10]. DENV has a genome of approximately 10,700 bases encoding a polyprotein that is cleaved to generate three structural proteins – core (C), precursor membrane (preM) and envelope (E) – and seven nonstructural (NS) proteins (NS1, NS2A, NS2B, NS3, NS4A, NS4B, and NS5)[11]. The open reading frame is flanked by two untranslated regions (5′ and 3′ UTRs), which are required for efficient translation and are highly conserved across flaviviruses [12]. Viral genome replication is error-prone due to the lack of proof-reading of the RNA-dependent RNA polymerase encoded by NS5, which results in a remarkable genetic and antigenic diversity within and across DENV serotypes[13]. Serotype-specific differences in host innate and adaptive responses, and ultimately pathogenesis, are incompletely characterized in part due to the scarcity of suitable experimental systems. While patient-derived viruses can establish infections in cell culture, their genetic heterogeneity impairs experimental reproducibility and complicates the interpretation of the resultant data. Doubtlessly, viral infectious cDNA clones offer a powerful, genetically defined tool for the characterization and manipulation of viral genomes for the downstream experiments. Full-length flavivirus infectious clones are notoriously difficult to propagate given their instability in bacteria[14]. Consequently, limited numbers of infectious clones – most of which are of serotype 2 - have been reported and thus hamper molecular characterization of differences across different DENV serotypes[15].

To address some of these challenges, we constructed 20 infectious clones by using circular polymerase extension reaction (CPER) to bypass recipient hosts (bacteria and yeast). We validated that these clones capture the antigenic complexity of DENV and replicate robustly in both mammalian and insect cell lines. To establish the potential utility of these clones for analyzing host responses to DENV infection, we characterized infection kinetics in a humanized mouse model. Humanized mice, animals engrafted with components of a human immune system, can be routinely generated by injecting human hematopoietic stem cells (HSCs) in suitable xeno-recipients[16]. HSC-transplanted mice were shown to be susceptible to DENV viruses, including clinical isolates[17] and to mimic several of the associated clinical features observed in dengue patients[18–20]. Here, we utilized a second-generation humanized mouse model in which human myeloid and natural killer (NK) cells can be selectively expanded promoting transcriptomic responses to the yellow fever virus 17D vaccine (YFV-17D) akin to those of human vaccinees[21]. Notably, representative infectious clones of all four genotypes cause infections in these humanized mice, triggering activation of T cell immunity and weak humoral immunity. Collectively, we describe here the creation and characterization of arguably the largest collection of DENV infectious clones, which we believe will be a valuable asset for studying DENV.

## Materials and methods

**Institutional approvals.** All animal experiments described in this study were performed in accordance with protocols (number 1930) that were reviewed and approved by the Institutional Animal Care and Use and Committee of Princeton University. All mice were maintained in facilities accredited by the Association for the Assessment and Accreditation of Laboratory Animal Care. All work with infectious DENV was reviewed and approved by the Institutional Biosafety Committee of Princeton University (protocol registration number 1060).

**Cells and antibodies.** Human hepatocellular carcinoma-derived Huh7.5 cells[22] (kindly provided by Dr. Charles Rice, The Rockefeller University), were maintained in Dulbecco’s modified Eagle’s medium (DMEM) that was supplemented with 10% heat inactivated fetal bovine serum (FBS, Atlanta Biologicals, Norcross, GA, USA) and 1% Penicillin Streptomycin (Pen/Strep, Thermo Scientific, Waltham, MA, USA) and kept at 37°C in a 5% CO_2_. The *Aedes albopictus* mosquito-derived cell line C6/36 (ATCC®CRL-1660™) was cultured at 28°C in Leibovitz's L-15 Medium (Thermo Fisher Scientific, Waltham, MA), and supplemented with Tryptose Phosphate Broth solution (Millipore Sigma, Burlington, MA, USA), 10% FBS and 1% Pen/Strep. The antibodies used in this study are summarized in **Supplementary Materials and Methods**.

**DENV reconstitutions and preparations.** The twenty clinical isolates used in this study (Table S1) were acquired from the World Reference Centre for Emerging Viruses and Arboviruses. The viruses were originally isolated from serum of infected patients and have been reconstituted with 1 milliliter of PBS and once propagated in Huh7.5 cells. Supernatants were collected from the infected cells, and the infectious titers were determined and expressed as focus forming unit (FFU) per milliliter. The virus was aliquoted and cryopreserved at –80°C until needed for subsequent use. The titer of our viral stocks and CPER-derived viruses was determined using a FFU assay (described in **Supplementary Materials and Methods).**

**Viral genome sequencing**. The detailed procedures are described in **Supplementary Materials and Methods**. All sequences have been deposited in the NCBI Gene Expression Omnibus (GEO) database (accession numbers OK605753 – OK605771, OL452067).

**Generation of infectious DENV clinical isolate clones by circular polymerase extension reaction (CPER).** The cDNA fragments from clinical isolates were cloned into pCR-Blunt II-TOPO vectors (Thermo Scientific, Waltham, MA). The CPER was conducted with some modification of the previous report[23]. The detailed procedures are found in **Supplementary Materials and Methods**. All plasmids are available upon request from the Ploss lab.

**Figure 1:**
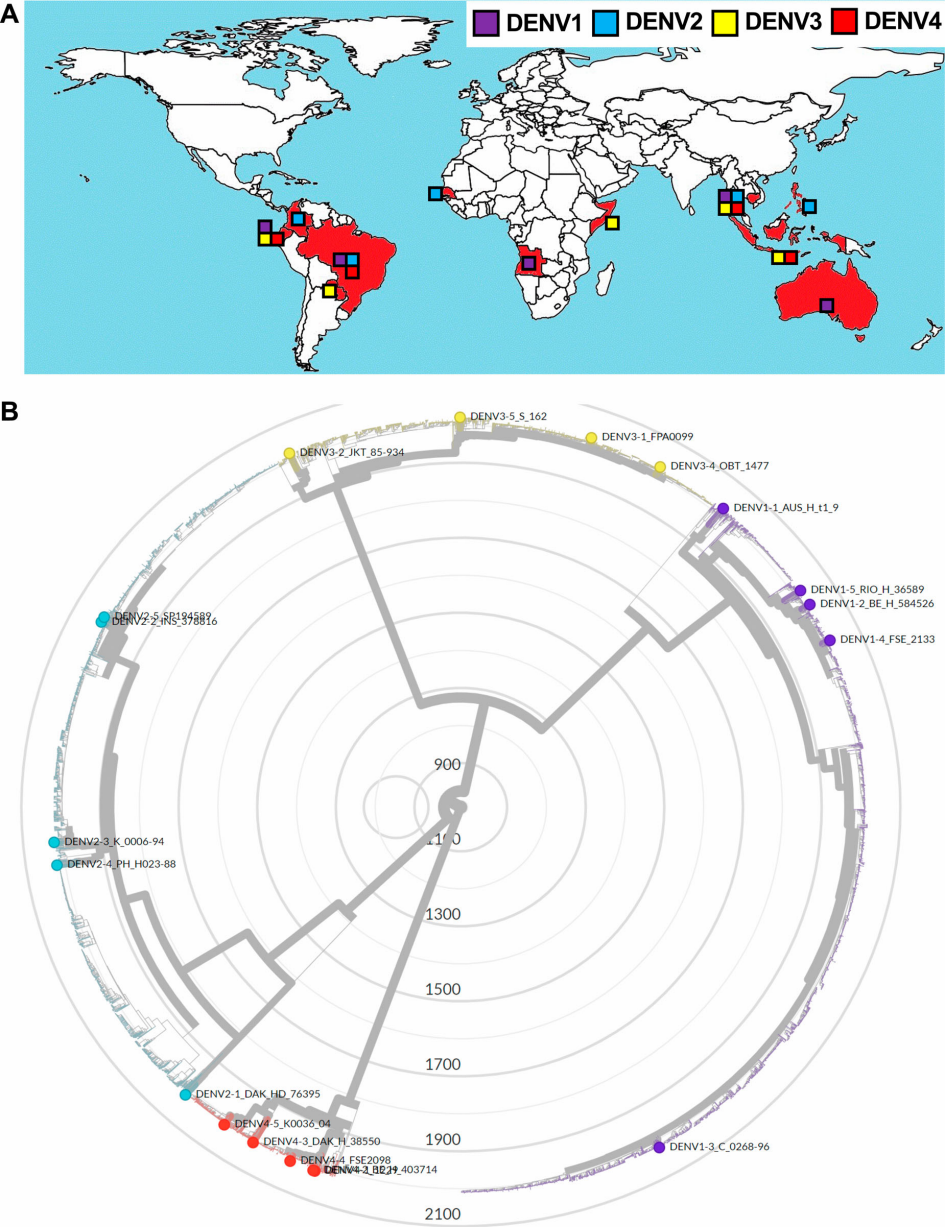
. **The 20 clinical isolates reflect the antigenic and geographic diversity of DENV**. (A) Geographic distribution of the 20 clinical isolates. Block charts reflect frequencies of different viral serotypes originating from respective geographic regions (DENV-1, purple; DENV-2, blue; DENV-3, yellow; DENV-4, red). (B) Phylogenetic tree constructed using full-genome sequences of 5,448 published DENV strains and reference strains obtained from NCBI GenBank. The 20 clinical isolates are plotted in different colors on basis of serotype and used to infer comparative evolutionary history. Tree was drawn using the maximum likelihood method and scale expresses genetic distance.

**Alignment of viral genomes and phylogenetic analysis.** To represent the evolutionary relationship among the 20 clinical isolates in the context of all known dengue strains found in humans, a phylogenetic tree was constructed (Figure 1B) by Nextrain phylogenetic tree construction[24]. The 3' and 5'UTRs of the 20 clinical isolates were aligned by MUSCLE formula[25]. The detailed analyses were summarized in **Supplementary Materials and Methods**.

***In vitro* virus replication kinetics.** *In vitro* growth kinetics of original clinical isolates and CPER-derived viruses were evaluated in susceptible cell lines and compared with growth properties. Huh7.5 or C6/36 cells were infected a multiplicity of infection (MOI) of 0.1 on the basis of viral titers previously determined in the Huh7.5 cells. Cellular RNA was extracted at indicated timepoints, and subject to RT-qPCR for generating viral growth curves. Culture supernatants at day 4 post-infection were harvested and tittered by FFU assay.

**RT-quantitative real time PCR.** For quantification of viral RNA copies, viral RNA was extracted from infected mouse serum or supernatants by using Zymo Viral RNA extraction kit (Zymo Research, Irvine, CA, USA) and from cells by using EZ-10 Spin Column Total RNA Mini-Preps Kit. Then first-strand cDNA synthesis and qRT-PCR were performed by using a qScript One-Step RT-qPCR Kit (Quantabio, Beverly, MA, USA) and StepOnePlus Real-Time PCR System (Thermo Scientific, Waltham, MA, USA), respectively, according to the manufacturer’s protocols. For quantification of viral RNA, the primer set for the detection of the noncoding region reported in the previous study was used[26]. Fluorescent signals were determined by the StepOnePlus Real-Time PCR System (Thermo Scientific, Waltham, MA, USA).

**Mouse models and housing.** NOD *Rag1*^−/−^ *IL2Rg^null^* (NRG) mice (NOD.Cg-*Rag1^tm1Mom^Il2rg^tm1Wjl^*^/SzJ^)[27] were obtained from The Jackson Laboratory (Bar Harbor, MA, USA; catalog number 007799). NRG-Flk2^–^/^–^ HLA-A*0201 (NFA2) mice (NOD.Cg-*Rag1^tm1Mom^ Flt3^tm1Irl^ Il2rg^tm1Wjl^*/J HLA-A*0201) were generated as described previously[21] and are available at The Jackson Laboratory (“catalog number 033127)”. NRG and NFA2 mice were maintained at the Laboratory Animal Resource Centre at Princeton University.

**Isolation of human CD34^+^ hematopoietic stem cells (HSC).** Human fetal livers (16–22 weeks of gestational age) were procured from Advanced Bioscience Resources (ABR), Inc. (Alameda, CA, USA). Fetal liver was homogenized and incubated in digestion medium, HBSS with 0.1% collagenase IV (Millipore-Sigma, Burlington, MA, USA), 40mM HEPES, 2M CaCl_2_ and 2U/ml DNase I (Roche, Basel, Switzerland) for 30 min at 37 °C. Human CD34^+^ HSC were isolated using a CD34^+^ HSC isolation kit (Stem Cell Technologies, Vancouver, Canada) according to the manufacturer’ protocol. Purification of human CD34^+^ cells were assessed by flow cytometry using an anti-human CD34^+^-FITC antibody (BD Biosciences, Franklin Lakes, NJ, USA).

**Generation of Adeno-associated virus (AAV) expressing human FLT3Lg.** The detailed methods are found in **Supplementary Materials and Methods**. AAV-293 cells (Agilent, Santa Clara, CA) were transfected via the calcium phosphate method. The purified AAV were analyzed via silver stain to check purity and qPCR to quantify.

**Generation of human immune system-engrafted mice.** 4–6 h prior to injection, one-three day old NFA2 mice were irradiated with 300 cGy. Following irradiation the mice were injected intrahepatically with 1.5–2 × 10^5^ human CD34^+^ hematopoietic stem cells to generate HIS-NFA2 mice. After 12 weeks, human hematopoietic chimerism was assessed in peripheral blood by flowcytometry. Mice with peripheral engraftment level >20% were enrolled in the study. HIS-NFA2 mice meeting this threshold were subsequently injected intravenously (tail vein) with 10^12^ AAV-hFlt3Lg 10 days prior to DENV infection. Male and female mice transplanted with CD34^+^ HSC derived from four different human donors were used in this study.

***In vivo* DENV infection experiments.** HIS-NFA2/hFlt3Lg mice were infected intravenously or subcutaneously with the doses specified in the figures. Mice were monitored daily for any clinical signs of disease. 200 µL of blood was collected through submandibular bleeding at the indicated timepoints. Serum was separated by centrifugation (10 min, 1,500 x *g* at room temperature) and used for RNA extraction, immune cell characterization, and enzyme-linked immunosorbent assays (ELISAs).

**Immune cell preparation, antibody staining and flow cytometry.** The detailed experimental procedures are found in **Supplementary Materials and Methods**. 2–4 × 10^6^ PBMCs were aliquoted per staining reaction and stained for 1 h at 4 °C in the dark with the appropriate antibody mixtures. After being washed with 1% (v/v) FBS in PBS, cells were fixed in the dark for 20 min at 4 °C with fixation buffer (1% (v/v) FBS, 4% (w/v) PFA in PBS). Flow cytometry was conducted using an LSRII Flow Cytometer (BD Biosciences) and data was analyzed using FlowJo software (TreeStar, Ashland, OR, USA).

**ELISAs.** Total human IgG and IgM antibodies were quantified using a human IgG or IgM ELISA quantitation set (Bethyl Laboratories, Montgomery, TX, USA). Human FLT3Lg concentrations were assessed using an in-house sandwich ELISA as the previously reported[21].

**Statistical Analysis.** Statistical analyses were performed using either a Student’s *t* test, Friedman, a one-way-ANOVA, a Wilcoxon signed rank, or a Mann–Whitney U test (GraphPad Prism software ver. 9.2.0) when appropriate and as indicated in each ﬁgure legend). *p ≤ 0.05.

## Results

***Low-passage DENV isolates from distinct geographic regions are genetically and antigenically diverse.*** Currently, few infectious DENV clones exist—hampering virological and immunological studies. To address this technical gap, 20 patient-derived clinical isolates covering strains from all four serotypes (Table S1), were obtained from the World Reference Centre for Emerging Viruses and Arboviruses. These viruses, originally isolated from the serum of infected patients, were selected as representatives of diverse geographical locations in 11 countries spanning South Asia, Southeast Asia, Australia, and South America **(**Figure 1a and Table S1). The isolates were amplified in a single passage in human Huh7.5 hepatoma cells **(**Supplementary Figure 1), a cell line highly permissive to various positive-sense, single stranded RNA viruses[22]. Viral RNA extracted from these culture supernatants was subjected to RNA sequencing, yielding high-quality genomic information for all but the 5’ and 3’ UTRs. Since the 5’ and 3’ UTRs contain genetic elements that are critical for viral replication and translation[28,29] their sequences were determined separately by viral RNA ligation for circularization of cDNA. Collectively, these efforts allowed us to assemble pristine full-length genome sequences for all 20 viruses (NCBI accession numbers OK605753 – OK605771, OL452067). DENV, with its inherently high error-rate in RNA genome replication, exhibits extensive genetic diversity within each of the four serotypes[13,30,31]. To assess the level of diversity captured by our subset of clinical isolates, a phylogenetic analysis was performed based on full-length genome sequences (Figure 1B). To contextualize genetically the clinical isolates used here, we retrieved all available full-length DENV sequences published on GenBank at time of writing and compared a total of 5,448 full-length sequences with the 20 clinical isolates. The resulting phylogenetic tree (Figure 1B) revealed that the patient-derived isolates were uniformly distributed in terms of both serotype and evolutionary history, with only two pairs of isolates sharing a clade. Results from this phylogenetic analysis were used to confirm geographic origin, highlighting the expansive diversity of these isolates in terms of both genetic and regional variation. All DENVs group into four genetically related but antigenically distinct 4 serotypes. Across the different serotypes, DENV differ genetically by 25–35%. Within the single serotype, there is 94–97% homology at the amino acid level (Table 1 and Table S2). As per the 20 strains, the observed amino acid homology in viral envelope E protein ranges from 63–77% between serotypes and 97–98% within each serotype (Table 1) that are correlated with the homology level in the viral polyprotein (Table S2), indicating that the 20 strains certainly reflect genetic diversity of DENVs. The UTR regions contain highly conserved elements that are essential for flaviviral RNA replication. These previously described structural features within the 5’ and 3’ UTRs were also observed in the 20 strains (Supplementary Figure 2). Taken together, the 20 strains reflect adequately their antigenic and genetic diversity of circulating DENVs.

**Table 1.** Amino acid differences across the viral E proteins.

	Percentage of homology																			
	1–1	1–2	1–3	1–4	1–5	2–1	2–2	2–3	2–4	2–5	3–1	3–2	3–3	3–4	3–5	4–1	4–2	4–3	4–4	4–5
DENV1-1	100.00	98.18	97.58	97.98	97.98	70.10	68.08	68.48	68.48	67.88	77.37	76.97	76.36	77.58	77.58	64.04	63.84	63.84	64.24	63.43
DENV1-2		100.00	97.17	99.60	99.60	70.30	68.28	68.69	68.69	68.08	77.17	76.77	76.16	77.37	77.37	63.84	63.64	63.64	64.04	63.23
DENV1-3			100.00	97.17	97.17	69.90	68.28	68.69	68.69	68.08	77.78	77.17	76.57	77.98	77.98	63.64	63.43	63.43	63.84	63.03
DENV1-4				100.00	99.60	70.30	68.28	68.69	68.69	68.08	77.17	76.77	76.16	77.37	77.37	63.84	63.64	63.64	64.04	63.23
DENV1-5					100.00	70.51	68.48	68.89	68.89	68.28	77.17	76.77	76.16	77.37	77.37	64.04	63.84	63.84	64.24	63.43
DENV2-1						100.00	94.55	94.55	94.55	94.34	68.48	67.88	68.28	68.48	68.69	64.65	64.65	64.44	64.65	64.44
DENV2-2							100.00	97.58	97.98	99.60	68.28	67.47	67.47	68.28	68.48	64.04	64.04	64.24	64.04	64.24
DENV2-3								100.00	98.18	97.37	68.08	67.27	67.47	68.08	68.28	64.24	64.24	64.44	64.24	64.44
DENV2-4									100.00	97.78	68.28	67.47	67.68	68.28	68.48	63.84	63.84	64.04	63.84	64.04
DENV2-5										100.00	68.28	67.47	67.47	68.28	68.48	64.04	64.04	64.24	64.04	64.24
DENV3-1											100.00	96.35	96.35	98.78	98.99	62.83	62.83	63.64	63.03	63.23
DENV3-2												100.00	97.57	96.96	96.96	62.63	62.63	63.43	62.83	63.03
DENV3-3													100.00	96.96	96.96	63.03	63.03	63.84	63.23	63.43
DENV3-4														100.00	99.39	62.83	62.83	63.64	63.03	63.23
DENV3-5															100.00	63.03	63.03	63.84	63.23	63.43
DENV4-1																100.00	99.80	97.37	99.19	97.37
DENV4-2																	100.00	97.17	98.99	97.17
DENV4-3																		100.00	96.57	98.38
DENV4-4																			100.00	96.57
DENV4-5																				100.00

The percentage of amino acid similarity compared with sequence of viral envelope E is shown.

**Figure 2:**
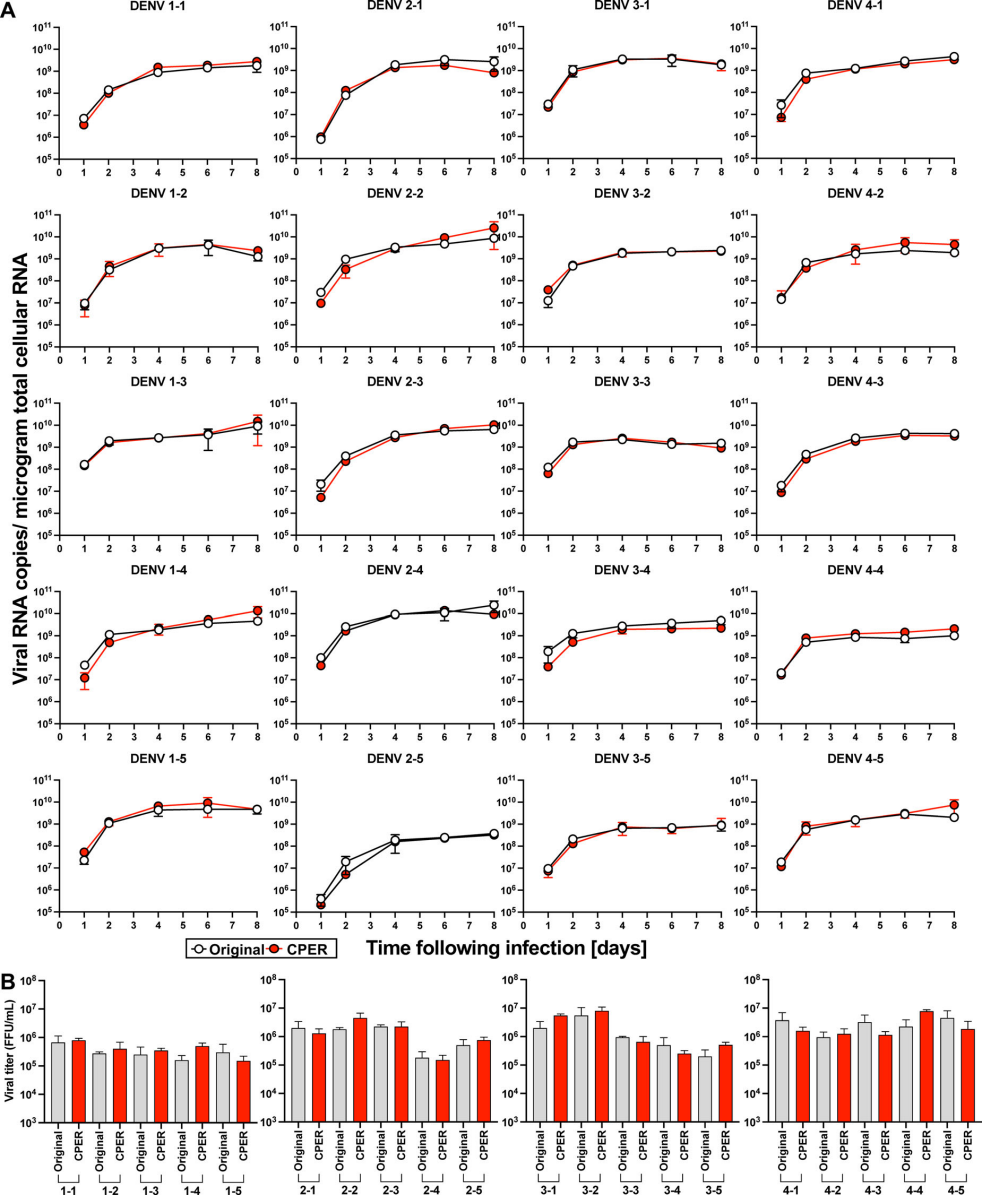
. **20 CPER-derived viruses mirror infection kinetics of the respective parental clinical isolates in human cell cultures.** Huh7.5 cells were infected with the parental clinical isolates and CPER-derived viruses at an MOI of 0.1. Intracellular viral RNA (A) was determined at the indicated timepoints by RT-qPCR, and viral titers (B) were quantified by a focus-forming assay at 4 days post infection. Statistical significance was assessed using Student’s *t* test (Original vs CPER).

**Figure 3:**
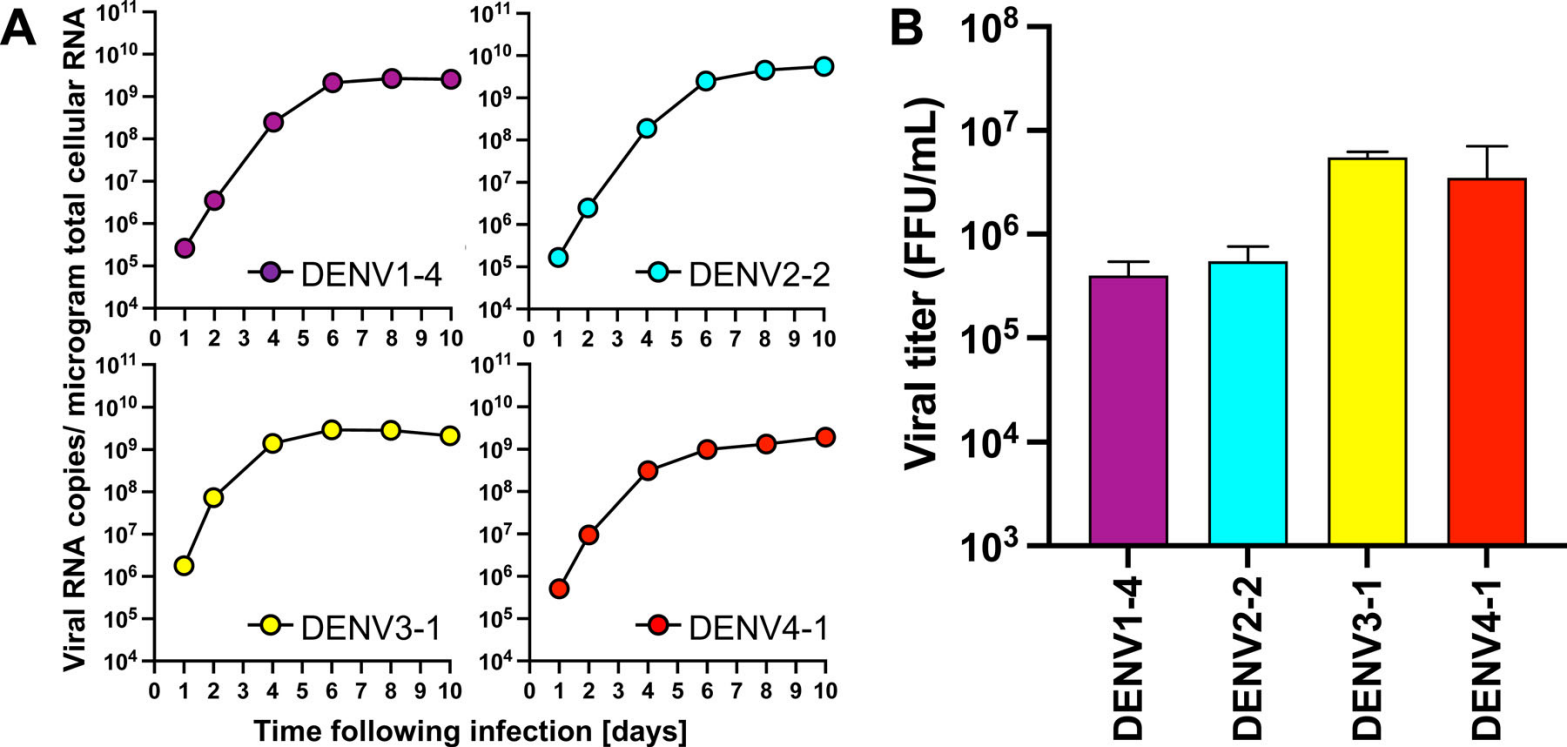
. **CPER-derived viruses infect and replicate robustly mosquito cells.** Mosquito-derived C6/36 cells were infected with the selected CPER-derived viruses from each serotype at an MOI of 0.1. Intracellular viral RNA (A) was determined at the indicated timepoints by RT-qPCR, and viral titers (B) were quantified by a focus-forming assay at 4 days post infection.

***Infectious clones covering DENV of all four serotypes replicate robustly in human and mosquito cell cultures.*** Having confirmed the genetic sequences and expansive diversity of the clinical isolates, infectious clones were generated *de novo* using circular polymerase extension reaction (CPER, Supplementary Figure 3)[32,33]. A full-length cDNA copy of the viruses offers a powerful tool for the characterization and manipulation of viral genomes – providing a genetically-defined population for research. However, cDNA encoding full-length genomes of flaviviruses are notoriously unstable and difficult to propagate in bacteria. Thus, we employed CPER, a method that does not require the generation of a DNA plasmid encoding the complete viral genome, accelerating the process of *de novo* virus generation. CPER has been used to successfully generate infectious flaviviruses including *e.g*. West Nile virus (WNV)[33], Zika virus (ZIKV)[34], Japanese encephalitis virus[35], and DENV[23]. Seven fragments covering each of the entire DENV genomes, in addition to a UTR linker, containing the human cytomegalovirus (CMV) promotor and a hepatitis delta virus ribozyme (HDVr), were amplified using PCR and cloned into individual pCR-Blunt II-TOPO vectors and their sequences validated by Sanger sequencing. Stocks for all 20 infectious clones were successfully launched following transfection of the CPER product into Huh7.5 cells. To compare the replicative capacity of the infectious clones with the parental viruses, we established growth curves in Huh7.5 cells (Figure 2). Notably, all 20 viruses replicated robustly in the human cells and there were no significant differences in the intracellular RNA copy numbers between the CPER-launched and the parental viruses for any of the strains (Figure 2A). Furthermore, both the parental and CPER-derived viruses produced similar viral titers ranging from 10^5^ to 10^6^ FFU/mL (Figure 2B), indicating that the CPER-derived viruses show robust propagation mirroring replication of the respective parental viruses in mammalian cell culture. Since DENVs are arthropod-borne viruses, we also established growth curves for these viruses in mosquito-derived cell line C6/36 (Figure 3). All of the CPER-launched viruses from each serotype exhibited a high infectivity in C6/36 cells. The intracellular viral RNA copy numbers increased over the first four days and plateaued after day 6 post-infection. The infectious titers on day 4 post-infection were similar to those of Huh7.5 cells, ranging from 10^5^ to 10^6^ FFU/mL. These results show that the infectious clones covering DENV of all four serotypes replicate robustly in mosquito cell cultures.

**Figure 4:**
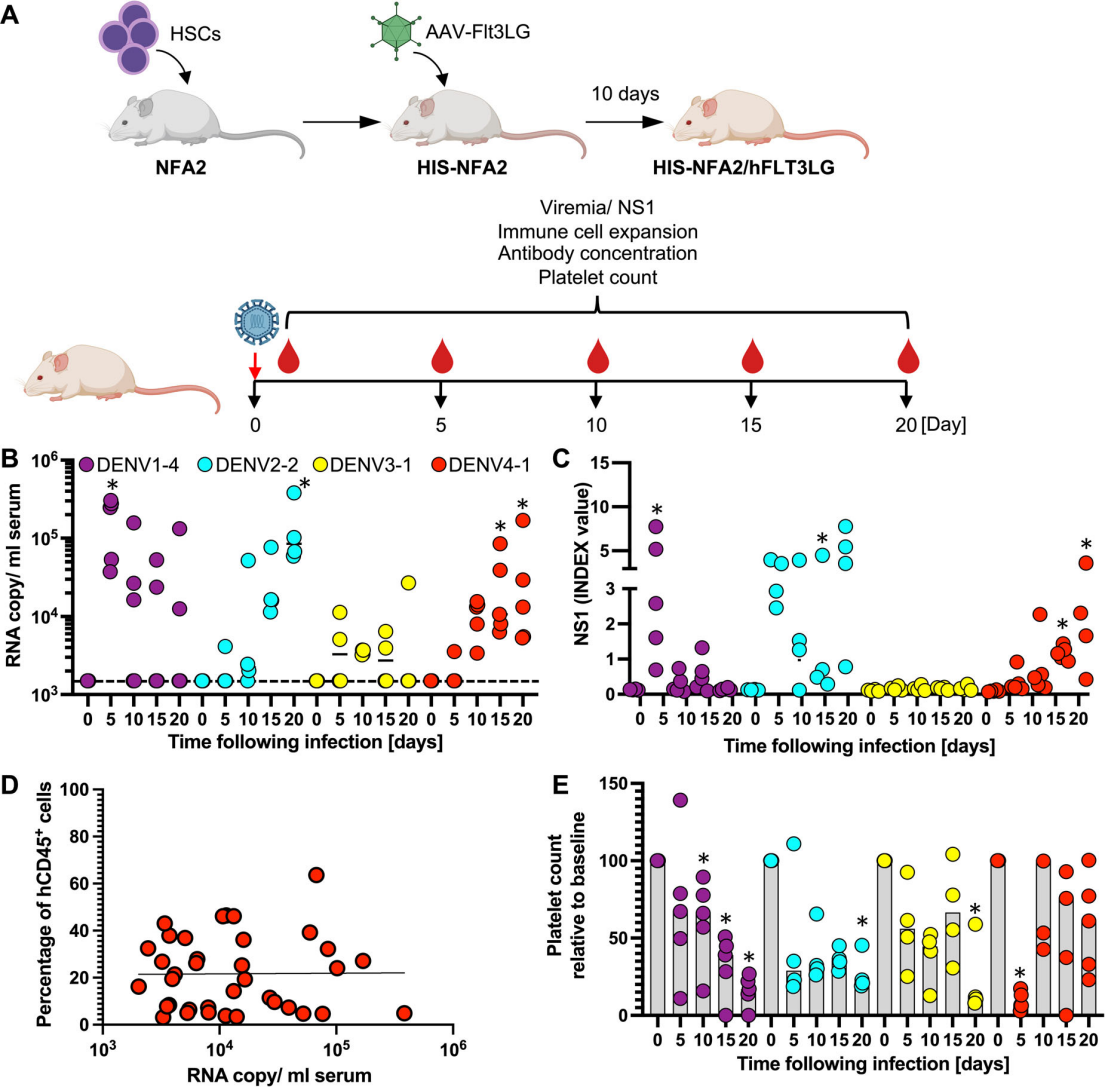
. **CPER-derived DENV of all 4 serotypes establish infection in humanized mice.** (A) Schematic overview for the generation of HIS-NFA2/hFLT3Lg mice utilized in the *in vivo* DENV experiments created with BioRender.com. (B) Four cohorts of mice were inoculated intravenously (i.v.) with 10^5^ FFU of either DENV1 (*n* = 5, purple), DENV2 (*n* = 4, cyan), DENV3 (*n* = 4, yellow), or DENV4 (*n* = 5, red). Mice were bled at indicated timepoints post-infection, and viral RNA was extracted from plasma and quantified by RT-qPCR. Results are presented as copies of viral RNA per milliliter of plasma measured until 20 days post-infection. The dashed line represents the threshold for viral RNA detection (1,500 RNA copies/ml). Each symbol represents one mouse. (C) NS1 antigen in plasma was quantified by ELISA. (D) Correlation between chimerism (number of human CD45^+^ cells divided by total CD45^+^ cells) and viremia. Blood samples from DENV1-4 infected HIS-NFA2/hFLT3LG mice were collected at days 5, 10, 15, 20. Samples were stained for hCD45^+^ and mCD45^+^ to determine the human hematopoietic chimerism and viral RNA copies were quantified in serum by RT-qPCR. Symbols represent individual mouse, and the straight lines are the linear regression plotted between chimerism and viral RNA copy numbers (Pearson’s r values of 0.000009725). (E) Human platelet counts were assessed longitudinally over the course of 20 days after DENV-infection. Mice from each of the 4 experimental cohorts were bled at the indicated timepoints. Samples were stained for hCD41a^+^ to assess the absolute cell count per microliter of blood. The numbers are normalized on the day of DENV- infection (baseline). The numbers are normalized on the day of DENV- infection (baseline). Statistical significance was assessed using Friedman with Dunnett’s test (B, C, E) and is indicated by asterisks (*, compared between data at day 0 or baseline).

***DENV 1–4 clones result in productive infection in humanized mice***. We next aimed to evaluate the replicative fitness of our DENVs *in vivo*. Given the limited replicative capacity of non-adapted DENV even in immunocompromised mice, we chose a second-generation humanized mouse model, HIS-NFA2/FLT3Lg mice, repopulated with components of a human immune system (HIS) following injection of human hematopoietic stem cells (HSCs). This model enables the selective expansion of human natural killer (NK) and various myeloid cell populations following administration of FLT3Lg, resulting in significantly more robust human immune responses to the yellow fever virus 17D (YFV-17D) vaccine than in conventional humanized mice[21]. In contrast to other commonly used murine models such as AG129 mice, humanized mice have previously been shown to support infection with non-adapted DENV [17]. We selected HIS-NFA2 mice with a human hematopoietic chimerism of at least 20% prior to FLT3Lg administration (Supplementary Figure 4). We developed an adeno associated viral (AAV) platform which resulted in robust and sustained FLT3Lg expression in NRG (Supplementary Figure 5A) and HIS-NFA2 mice (Supplementary Figure 5B). In line with our previous observations in HIS-NFA2, in which we expressed FLT3Lg using a replication incompetent adenovirus (AdV) [21], AAV-mediated FLT3Lg expression also led to selective expansion of human dendritic cells (DCs) and NK cells (Supplementary Figure 5C**).** We first compared different routes of DENV infection and inocula using the DENV-2 strain INS378816. Irrespective of whether DENV-2 was administered intravenously (i.v.) or subcutaneously (s.c.) the selected DENV-2 strain resulted in detectable viremia by day 10 post-infection (Supplementary Figure 6). Infections with 10-fold higher inocula (7 × 10^5^ vs 7 × 10^4^ FFU/mouse) resulted in slightly higher viremia in HIS-NFA2/FLT3Lg mice (Supplementary Figure 6). For all subsequent experiments we chose the higher dose delivered through the intravenous route, in order to increase our chances of successfully establishing infections with different serotypes. Given the complexity of the system, we limited our analysis to representative strains of all four DENV serotypes (DENV-1 FSE2133, DENV-2 INS378816, DENV-3 FPA0099, DENV-4 1229) for the proof-of-concept work described here (Figure 4A). Infections with viruses of all four serotypes resulted in viremia, however, the virus kinetics differed among the chosen viruses. Upon infection with DENV1–4, all 5 animals showed evidence of infection by day 5 post-infection, but the high RNA copy numbers and NS1 amounts declined in the majority of animals over time (Figure 4B and C). In contrast, infections with DENV-2 and −4, viral RNA copies and NS1 amounts rose in all animals over time and reached maximum levels by day 20. Infections with DENV-3 were arguably the least robust, albeit viremia could be detected by RT-qPCR at 10–50-fold lower levels compared to the other serotypes. Presumably, due to the low infection NS1 levels did not increase significantly over background viral RNA copies. Numerous previous studies demonstrated that human cells are a reservoir for DENV replication in patients[36–40] and humanized mice[17,19]. However, here viremia did not correlate with the human hematopoietic chimerism on day 20 post infection (Figure 4D). Decreases in viremia observed in some animals also cannot simply be explained by loss of the human graft as the frequencies of human CD45^+^ remained largely stable over time (Supplementary Figure 7). None of the animals developed any signs of clinically apparent disease, and body weight and temperatures (data not shown) remained stable across all cohorts over time. Given that thrombocytopenia is a hallmark of severe DENV infection[41], and we quantified the numbers of human and mouse platelets over time. Human platelet frequencies were low but already detectable prior to infection (data not shown). During the course of the infection human platelets were significantly lower for all DENV-infected mice (Figure 4E). In contrast, mouse platelet numbers were not significantly affected by the infection (Supplementary Figure 8). Collectively, these data show that the DENV infectious clones can establish infection in HIS-NFA2/Flt3Lg mice, albeit with variable efficiency.

**Figure 5:**
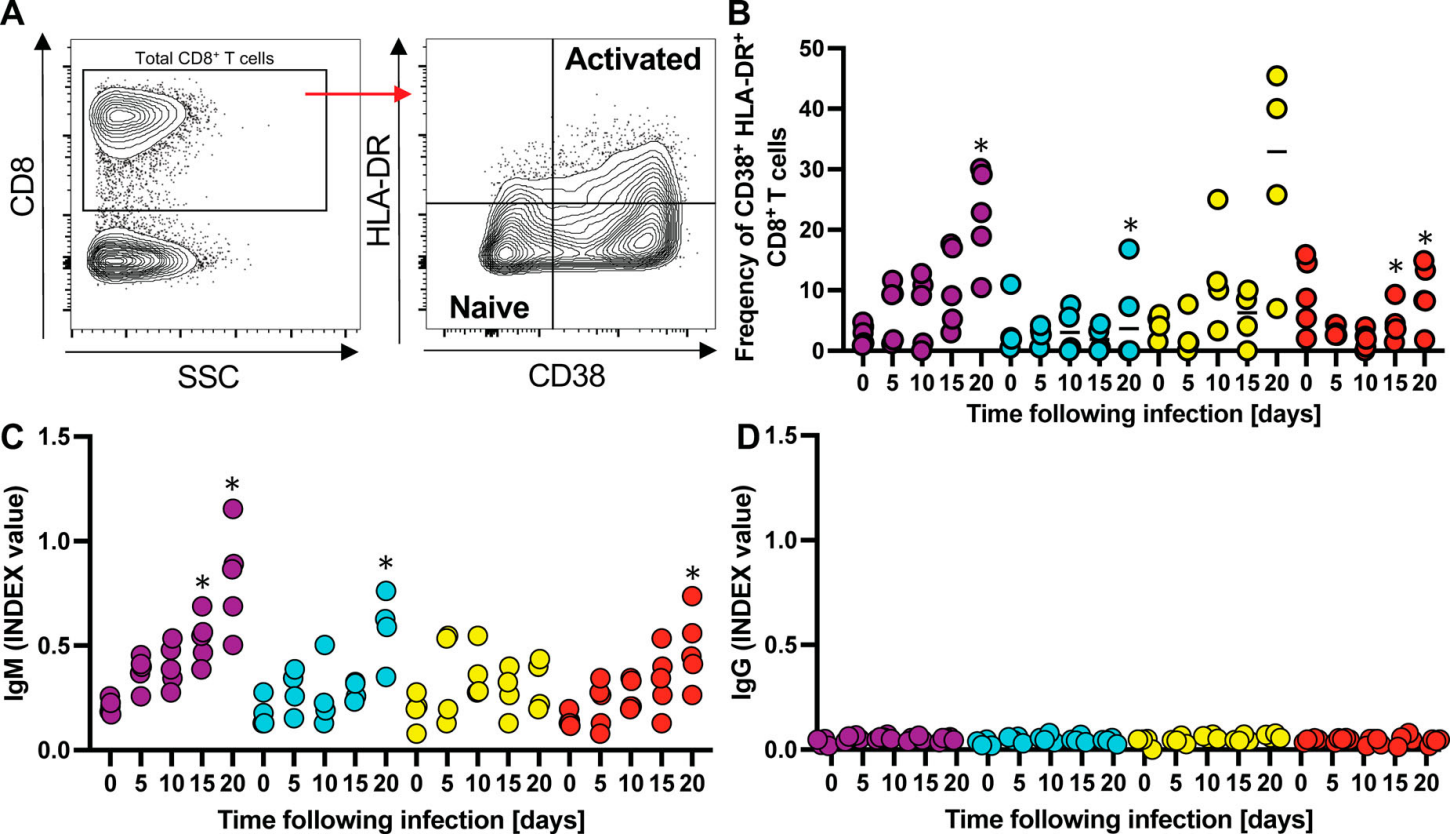
. **Infection of humanized mice with DENV results in activation of T cells and immune responses.** (A) Representative flow cytometry plots of CD38/HLA-DR gating, along with associated phenotypes. CD38 and HLA-DR expression was assessed among gated human CD8^+^ T-cells, and subsets were characterized as naïve (CD38^–^ HLA-DR^–^) or activated (CD38^+^ HLA-DR^+^) [43]. Frequencies of the activated subpopulation (CD38^+^ HLA-DR^+^) within CD8^+^ T cells in the plasma (B) are shown. All data is reported as frequencies of total CD45^+^ cells. Human DENV-specific IgM (C) IgG (D) antibodies circulating in sera of HIS-NFA2/hFLT3Lg mice infected with DENV. Both antibody levels were assessed using ELISA at days 0, 5, 10, 15, and 20 post-infection. Symbols represent individual mice and the median presented as a line per subset. Statistical significance was assessed using Friedman with Dunnett’s test (B, C, and D) and is indicated by asterisks (*, compared between data at day 0 or control cohort).

Next, we started to characterize the human immune response against the different DENVs. CD8^+^ T cells, in particular, play a key role given their implications in both protective and pathological effects against DENV[42]. Given the importance of this immune cell subset, we recorded the kinetics of activated CD8^+^ T-cells in DENV-infected mice. The frequencies of CD8^+^ T-cell activation were determined using surface staining for CD38 and HLA-DR - two cell surface markers that have been previously used to track virus-activated cells over time. Naïve cells are characterized by the CD38^–^ HLA-DR^–^ phenotype, while activated cells have a CD38^+^ HLA-DR^+^ phenotype (Figure 5A). In the DENV infected mice the frequencies of CD38^+^ HLA-DR^+^ CD8^+^ T cells steadily increased over time reaching 5–30% across the different experimental cohorts by day 20 (Figure 5B). These observations are consistent with the frequencies of CD38^+^ HLA-DR^+^ CD8^+^ T cells in YFV-17D and smallpox vaccinees day 15 after immunization[43]. Notably, the numbers of CD38^+^ HLA-DR^+^ CD8^+^ T cells were most elevated in the DENV-3 infected HIS-NFA2/FLT3Lg which also exhibited the lowest viremia (Figure 4B), suggesting that this virus might have been cleared by a more robust cellular immune response. We then aimed to quantify DENV-specific IgM and IgG levels to gain some insights into the humoral immune responses against the different serotypes (Figure 5C). Virus-specific IgM were slightly elevated in the mice infected with DENV1, 2, 4 but not detectable in mice infected with DENV3, correlating with lower viremia (Figure 4B). In contrast, DENV-specific IgG remained at background levels for all 4 cohorts on day 20 post-infection, (Figure 5D). These data, particularly the lack of evidence for antibody class-switching, is consistent with previous characterizations of humoral immunity against DENV and other viruses in humanized mice[17,44,45]. Altogether, these data indicate that the different DENV serotypes can establish infections in HIS-NFA2/hFLT3Lg mice, albeit with different efficiencies. Infections induced both cellular and humoral adaptive immune responses. Future studies will focus on potential differences in innate and adaptive immune responses including a more detailed characterization of cellular immunity. In particular, quantification and phenotypical and functional analysis of DENV-epitope specific CD8^+^ T cells using MHC multimers which may differ across the different DENV serotypes.

## Discussion

Here we lay out a general workflow for rapidly determining whole sequences of DENV clinical isolates and for generating DENV infectious clones from patient samples. The procedures detailed herein are applicable for characterizing genetically and for analyzing the replicative fitness of viruses derived from other clinical specimens *in vitro* and *in vivo*. This protocol can likely also be applied to the generation of infectious clones for other positive sense RNA viruses. We describe the generation of 5 genetically defined infectious clones for each of the four DENV serotypes. The 20 strains used in this study were genetically characterized and show expansive diversity of these isolates in terms of both genetic and regional variation. This is of particular note given that strains from different regions may vary in virulence, transmission, and disease severity. Establishing a repository of clinical isolates that reflect this variation allows for subsequent virological characterization and comparative analyses. In addition, we demonstrate that the representative strains from each of the 4 serotypes were able to replicate both in mammalian and mosquito cell cultures, as well as in the humanized mice.

Because of the instability of flavivirus genome in bacteria[14], limited clones have been accessible thus far – most of which are of serotype 2 (reviewed in [15]). Of the few that have been published, an even smaller number of DENV infectious clones are based on low passage isolates. An emphasis was placed on low-passage viruses given that mutations tend to arise upon sequential passaging in biological systems. Thus, in our study, we utilized DENV clinical isolates that were passaged no more than three times in C6/36 cells—striving to resemble more closely the original genotypic and phenotypic characteristics of circulating DENV strains. Upon the genetic characterization of the 20 strains, we efficiently established a library of DENV infectious clones using the circular polymerase extension reaction (CPER) method[32] to bypass propagation of flavivirus genomes in bacteria/yeast previously used to establish infectious clones of flaviviruses[23,33–35]. Our study demonstrates that this approach can be utilized to efficiently construct full-length DENV infectious clones based on low-passage clinical isolates. A diverse library of 20 genetically defined infectious clones was subsequently generated and these *in vitro* characteristics proved to mirror the parental clinical isolates. In addition, our full-length DENV infectious clones might also be useful for diagnostic and neutralization assays using patient-derived serum since the clones reflect adequately the antigenic diversity of circulating DENVs.

The paucity of experimental models that faithfully recapitulate the various aspects of DENV disease continues to pose a major barrier towards the study of this viral disease. The emergence of humanized mice, engrafted with human tissues and/or expressing human genes, offers great potential for the study of human-tropic pathogens such as DENV. In this study, we employed a second-generation humanized model termed as HIS-NFA2/hFLT3Lg[21]. Our prior work has established that HIS-NFA2/hFLT3LG mice mount an immune response to YFV-17D that is highly akin to that seen in humans[21]. Additionally, this system allows for a more robust expansion of human NK and various myeloid cells[21,46]—components of the immune system that are particularly important in the pathogenesis and immune response elicited by DENV. Upon *in vivo* challenge using our patient-derived CPER viruses, infected HIS-NFA2/hFLT3LG mice showed persistent viremia, with levels reaching up to 4 × 10^5^ viral RNA copies per milliliter. These levels of viremia are comparable to the previous reports utilized humanized mice[18,20]. The establishment of viremia is a key clinical aspect of DENV disease that is replicated by infected HIS-NFA2/hFLT3Lg mice. In humans, DENV causes acute infection and viremia is resolved thereafter within a week[47]. By contrast, we detected viremia well beyond 20 days post-infection. This prolonged period of viremia has also been reported in other humanized mice studies, suggesting that poor viral clearance could be due to significantly low DENV-specific IgG antibody responses—a drawback shared by many current humanized mice models[17,44,45,48]. Consistent with our other studies utilizing intravenous inoculation, a less natural route of administration, our findings also demonstrate that this i.v. route yielded the highest levels of viremia in HIS-NFA2/hFLT3Lg mice. Subcutaneous infection, considered to best mimic the bite of an infected mosquito, established viremia at slightly lower levels that was correlated with DENV titers. To increase our chances of successfully establishing infections with selected strains from the four serotypes, in all subsequent experiments we chose the higher dose delivered intravenously. Thus, the optimal dose and route of inoculation likely varies depending on the clinical isolate that is being used. Different combinations of infection dosage and administration route can also be tested to assess more optimal experimental conditions for this particular clinical strain.

Having established this baseline analysis, we were able to more confidently associate subsequent changes in human platelet levels with DENV infection. Particularly when infected with DENV1 FSE2133 strain, HIS-NFA2/hFLT3LG mice display thrombocytopenia that is specific to human platelets despite the high prevalence of murine platelets. This is in contrast to other established humanized mice that either display a drop in total platelet counts, or do not exhibit any changes in platelet frequencies[17,19,20]. This induction of human-specific thrombocytopenia—a prominent clinical manifestation of severe DENV—confers an important advantage to our model. Prospective clinical studies have highlighted the correlation between viremia and degree of DENV disease severity[49]. Given the prevalence of thrombocytopenia among dengue fever patients, experimental systems like HIS-NFA2/hFLT3Lg may also be used to explore the underlying mechanisms behind this pathology.

T-cells are major players governing the immune response against DENV[42]. We observed increased levels of CD8^+^ T-cell activation, as tracked by CD38 and HLA-DR markers, particularly within humanized mice infected with all DENV-infected cohorts. This enhanced T-cell activation correlates with the viremia levels observed in HIS-NFA2/hFLT3LG mice when infected with DENV-1, -2, -4, but not DENV-3. Such trends have also been described in clinical settings, as patients displayed a massive expansion of HLA-DR+ CD38^+^ CD8^+^ T-cell subsets upon DENV infection[50]. These markers, indicative of antigen-stimulation, tissue homing, and cytotoxic-effector function, provide support for the enhanced T-cell activation in DENV-infected HIS-NFA2/hFLT3Lg mice. Notably, however, the activation of these cytotoxic T lymphocytes was not associated with better viral control as seen in clinical settings[51,52]. This could potentially be due to improper T-cell priming as a result of inadequately organized lymphoid organs. Similar to the humoral response reported in previous humanized mice, HIS-NFA2/hFLT3LG mice also mounted specific IgM but not IgG antibodies to DENV infection[17,44,45]. The lack of IgG antibodies largely reflects the general inability of current humanized mice to support immunoglobin class switching. This may be attributed to a number of factors including the poor development of follicular structures, inadequate activating human cytokines, and high levels of immature B cells in the periphery[16,53]. This deficient humoral response marks a significant limitation in current versions of humanized mice, especially given the postulated roles of antibodies in both the pathogenesis and neutralization of DENV. Further immunological improvements are required in humanized mice to better characterize these key DENV-mediated processes *in vivo*.

In summary, the library of 20 full-length infectious clones based on DENV clinical isolates serve as a powerful tool for future *in vitro* and *in vivo* analyses, and the experimental animal model described herein holds promising potential for future *in vivo* characterization of patient-derived viral strains. The continued refinement of this model, particularly in terms of improving humoral response, can yield important insights regarding human(-tropic) pathogens leading to elucidate pathogenesis, serve as pre-clinical models, and ultimately work synergistically towards the eradication of dengue.

## Supplementary Material

Supplemental Material
