# Supplementary material for: Generation and characterization of genetically and antigenically diverse infectious clones of dengue virus serotypes 1–4
Source: Emerg Microbes Infect. 2022 Jan 7;11(1):227–39. doi: 10.1080/22221751.2021.2021808 (PMC8745371; doi:10.1080/22221751.2021.2021808)
Supplement: Supplemental Material [file TEMI_A_2021808_SM9943.docx]

**SUPPLEMENTARY INFORMATION**

**FOR**

**Generation and characterization of genetically and antigenically diverse infectious clones of dengue virus serotypes 1-4**

Tomokazu Tamura^1^, Jiayu Zhang^1^, Vrinda Madan^1¶^, Abhishek Biswas^1,2^, Michael P. Schwoerer^1^, Thomas R. Cafiero^1^, Brigitte L. Heller^1^, Wei Wang^3^, Alexander Ploss^1*^

^1^ Department of Molecular Biology, Princeton University, Princeton, NJ, USA

^2^ Research Computing, Office of Information Technology, Princeton University, Princeton, NJ, USA

^3^ Carl Icahn Laboratory, Lewis-Sigler Institute for Integrative Genomics, Princeton University, Princeton, NJ, USA

^¶^ Present address: Johns Hopkins University School of Medicine, Baltimore, MD, USA

*Correspondence: Alexander Ploss, PhD: aploss@princeton.edu

**CONTENT:**

**1. Supplementary Materials and Methods**

**2. Supplementary Figures**

**Supplementary Figure 1.** Experimental workflow of the present study.

**Supplementary Figure 2.** Nucleotide alignment of the 20 clinical isolates of DENV. **Supplementary Figure 3.** Gating strategy for quantifying human and mouse immune cell subsets in humanized mice.

**Supplementary Figure 4.** Human hematopoietic reconstitution of the HIS-NFA2 mice used in this study prior to AAV-FLT3Lg treatment.

**Supplementary Figure 5.** AAV-mediated expression of hFLT3LG results in expansion of human myeloid and natural killer cells in HIS-NFA2 mice.

**Supplementary Figure 6.** Comparison of the impact of inocula and routes of administration on DENV viremia in HIS-NFA2/hFLT3Lg mice.

**Supplementary Figure 7.** Ratio of human CD45+ cells in HIS-NFA2/hFLT3LG mice during DENV infection.

**Supplementary Figure 8.** Murine platelets remain largely stable during DENV infection.

**3**. **Supplementary Tables**

**Supplementary Table 1.** Information of the Dengue viruses used in this study.

**Supplementary Table 2.** Amino acid differences of the viral polyprotein.

**Supplementary Table 3.** Primer sets utilized for PCR amplicons.

**Supplementary Table 4.** Primer sets utilized for PCR of circular cDNAs.

**Supplementary Table 5.** Primer sets utilized for CPER.

**4**. **Supplementary References**

**SUPPLEMENTARY MATERIALS AND METHODS**

**Antibodies and reagents.** The following anti-mouse Abs were used: From Biolegends (San Diego, CA, USA): CD45-PE-Cy7 clone 30-F11 (dilution 1/100); From Thermo-scientiﬁc/eBiosciences (Waltham, MA, USA/San Diego, CA, USA): CD41a-FITC clone HIP8. The following anti-human Abs were used for flow cytometry: From BD Biosciences: CD45-V500 clone HI30 (dilution 1/50), CD19-allophycocyanin-Cy7 clone SJ25C1 (dilution 1/100), CD8-FITC clone G42-8 (dilution 1/50), CD11c-APC clone B-ly6 (dilution 1/50), CD34-FITC clone 581 (dilution 1/100), CD90-PE clone 5E10 (dilution 1/100), CD38-PerCP-Cy5.5 clone HIT2 (dilution 1/100), CD45RA-allophycocyanin clone HI100 (dilution 1/100), HLA-A2-FITC clone BB7.2 (dilution 1/100), HLA-DR-FITC G46-6 (dilution 1/100); From Life Technologies, Invitrogen (Carlsbad, CA, USA); CD3-PE-Cy5 clone 7D6 (dilution 1/50), CD16- PE-Texas Red clone 3G8 (dilution 1/50), CD19-Pacific Blue clone SJ25-C1 (dilution 1/50); From Biolegends: CD56-allophycocyanin-Cy7 clone HCD56 (dilution 1/100); From Thermo-scientiﬁc/eBiosciences: CD3-allophycocyanin-eFluor780 clone UCHT1 (dilution 1/100), , CD4-PE clone RPA-T4 (dilution 1/50), CD14- Alexa Fluor 700 clone TuK4 (dilution 1/50), HLA-DR-eFluor450 clone L243 (dilution 1/100), CD123-eFluor450 clone 6H6 (dilution 1/50), CD19-PE-eFluor610 clone HIB19 (dilution 1/100), CD41a-allophycocyanin clone eBioMWReg30 (dilution 1/20); From Miltenyi Biotec (Cambridge, MA, USA): CD303/BDCA-2allophycocyanin clone AC144 (dilution 1/50). The anti-Mouse antibody D1-4G2-4-15 clone 4G2 (Novus Biological, Littleton, CO, USA) for FFU assay (dilution 1/1000) visualizing DENV viral protein E. LIVE/DEAD Fixable Blue Dead Cell Stain Kit (Thermo Scientific, Invitrogen) was used to determine the viability of cells for flow cytometry.

**Titration of DENV.** The titer of our viral stocks and CPER-derived viruses was determined using an FFU assay. Huh7.5 cells were seeded into a 48-well plate at a density of 1.0 × 10^4^ cells per well and infected the next day with 10-fold serial dilutions of the virus stock for 1 h at 37 °C. Following virus adsorption, 0.5 ml of overlay media overlaid with culture medium supplemented with 1% methylcellulose and 10% FBS was added in each well. Four days later, the overlay medium was removed, the cell monolayer was washed with 1X PBS for three times and fixed for 30 min at room temperature with 4% paraformaldehyde (PFA: Millipore Sigma, Burlington, MA, USA). Fixed cells were then washed with 1X PBS and stained with anti-E antibody and visualized with a DAB Substrate Kit, Peroxidase (HRP), with Nickel, (3,3'-diaminobenzidine) (Vector Laboratories, Burlingame, CA USA). Number of foci number were counted and expressed as FFU per milliliter.

**Viral genome sequencing**. Viral RNA (1 µg) from each clinical isolate obtained from the infected Huh 7.5 cells was subject to reverse transcription using serotype-specific primers listed in **Table S3**. This resulted in the synthesis of cDNA that was then subject to PCR for amplification using PrimeSTAR GXL DNA Polymerase (Takara Bio, Shiga, Japan). The RT-PCR products from viral samples were turned into sequencing libraries using the Nextera™ DNA Sample Prep Kit (Illumina, CA, USA) with different DNA barcodes attached to each library. The libraries were examined on Bioanalyzer DNA High Sensitivity chips (Agilent, CA) for size distribution, and quantified using a Qubit fluorometer (Invitrogen, CA, USA). Each set of libraries was pooled at equal molar amounts and sequenced on a MiSeq v2 Flowcell (Illumina, CA) as pair-end 2x250 nt reads. Only the Pass-Filter (PF) reads were used for further analysis. After trimming the Illumina adapter sequences, the pair-end reads from each sample were processed through the Unicycler (version 0.4.8.0) assembly pipeline[1] to obtain the viral genome assembly. Additionally, the sequences of the 5' and 3' UTRs were determined to enhance accuracy of DENV clone generation. RNA was extracted from the original virus stock using Zymo Quick-RNA viral kit (Zymo Research, Irvine, CA, USA) and was denatured at 65°C for 5 mins. The viral RNA was then decapped using Tobacco Decapping Enzyme (Enzymax, Lexington, KY, USA) and purified using the Zymo RNA Clean & Concentrator kit (Zymo Research, Irvine, CA, USA). Purified RNA was subject to RT-qPCR using SuperScript IV Reverse Transcriptase (Thermo Scientific) and Q5 polymerase (New England Biolabs, Ipswich, MA, USA) with primer sets listed in **Table S4**. The following PCR cycle was used: 98°C for 30 seconds, followed by 35 cycles of 10 s at 98°C and 15 s at 72°C, and a final extension at 72°C for 2.5 min. The gel-purified PCR product was then sent for sequencing by the Sangar method (Eton Bioscience, San Diego, CA). The sequencing data is visualized using SnapGene ver. 5.3.2 (GSL Biotech, Albuquerque, NM, USA). All sequences have been deposited in the NCBI Gene Expression Omnibus (GEO) database (accession numbers OK605753 - OK605771).

**Plasmids.** For each of the isolates, seven cDNA fragments spanning the entire viral genome were generated by PCR with the primer sets listed in **Table S5** and individually cloned into pCR-Blunt II-TOPO vectors (Thermo Scientific, Waltham, MA). An additional fragment encoding the polyA signal, cytomegalovirus (CMV) promotor, and hepatitis delta virus ribozyme (HDVr) site was cloned into an eighth pCR-Blunt II-TOPO vector. The constructed plasmids were verified by Sanger sequencing (Eton Bioscience, San Diego, CA). All plasmids are available upon request from the Ploss lab.

**Generation of Infectious DENV Clinical Isolate Clones by circular polymerase extension reaction (CPER).** Upon confirmation of plasmid sequences, eight PCR fragment sequences were generated Q5 polymerase and primer pairs that have complementary ends with a 25-nucleotide overlap listed in **Table S5**. The resulting eight DNA fragments were mixed in equimolar concentrations (0.1 pmol each) to generate circular DNA by CPER using PrimeSTAR GXL DNA polymerase. The following PCR cycling was used: 98°C for 2 minutes, followed by 20 cycles of 10 s at 98°C, 15 s at 55°C, 12 min at 68°C, and a final extension at 68°C for 12 min. The CPER products were then transfected into Huh7.5 cells using the X-tremeGENE™ HP DNA Transfection Reagent (MilliporeSigma, Burlington, MA, USA). At 7- and 10-days post-transfection, the culture supernatants were collected, and viral titers were determined by FFU assay. Upon observation of the cytopathic effect (CPE), culture supernatant was harvested to recover newly generated *de novo* viral strains. Virus containing supernatant was aliquoted and cryopreserved at -80°C prior to use in future experiments.

**Alignment of viral genomes and phylogenetic analysis.** To represent the evolutionary relationship among the 20 clinical isolates in the context of all known dengue strains found in humans, a phylogenetic tree was constructed (**Fig. 1B**). We downloaded all full length DENV sequences from the NCBI Virus Variation database that were annotated to be found in human hosts as of 21^st^ February 2021. The nucleotide sequences and metadata information such as serotype and country of origin of the 5448 query results were downloaded. The sequence and metadata were formatted to produce a tree using the Nextrain phylogenetic tree construction and visualization tool [2]. To study the sequence variation in the 3' UTRs and 5' UTRs of the 20 clinical isolates, the nucleotide sequences were translated using the correct frame and the regions of interest were extracted. The 3' UTRs and 5' UTRs were grouped together into separate FASTA files and aligned using MUSCLE aligner[3] and the multiple sequence alignments were plotted using ClustalX2[4]. Additionally, to compute the pairwise protein sequence homology of various regions among the 20 strains the extracted regions were aligned using MUSCLE and the homologous amino acids counted, and the percentages computed.

**Generation of Adeno-associated virus (AAV) expressing human FLT3Lg.** The pAAV-EF1alpha-DIO-eNpHR-3.0-eYFP backbone containing AAV2 ITRs (Addgene, catalogue #26966) was digested with MluI HF/EcoRI HF. The CMV-Flt3 was PCR amplified from a pShuttle-CMV-Flt3 plasmid containing the Flt3 insert from pAL119-Flt3L (Addgene, catalogue #21910). The PCR product of the CMV-Flt3 had 15 bp of overlapping sequence with the backbone and was assembled with In-Fusion (Takara Bio, Shiga, Japan) to create the final construct. AAV-293 cells (Agilent, Santa Clara, CA) at 50% confluency in 15 cm dishes were transfected via the calcium phosphate method with 22.5 µg XR8 (NGVB, Indianapolis, IN, USA), 7.5 µg pHelper (Agilent, Santa Clara, CA), 7.5 µg of pAAV-CMV-Flt3-WPRE-hGH per plate. Media was collected every 24 hours for 72 hours total. After 72 hours, the media was treated with a 5x solution of 40% PEG8000 and 2.5 M NaCl to precipitate the AAV for two hours at 4°C before being spun down at 4300 x *g* for 20 minutes. Cells from plates were scraped, washed with PBS, and resuspended in hypotonic buffer (10 mM HEPES, 1.5 mM MgCl_2_, 10 mM KCl, 0.35 mg/ml spermine) on ice for 10 min before 1 ml restore buffer (62.5% sucrose wt/vol in hypotonic buffer) was added. Cell membranes were sheared in a 15 ml Kontes dounce homogenizer and nuclei were spun down at 500 x *g* for 10 min. AAV from PEG precipitate was resuspended in 6 ml high salt buffer (2.5 mM KCl, 1 mM MgCl_2_, 1 M NaCl in PBS) and added to nuclei that had been resuspended in 1 ml low salt buffer (2.5 mM KCl, 1 mM MgCl_2_, in PBS). Lysate was treated with 250 units of Benzonase (MilliporeSigma, Burlington, MA, USA) at 37°C for 30 min and then spun at 4300 x *g* for 30 min before being loaded onto an iodixanol gradient. AAV was spun at 38,000 rpm in an SW41 rotor for 3 hours at 16C. AAV was collected from the 40% iodixanol layer and buffer was exchanged to AAV storage buffer (PBS with 35 mM NaCl, 0.002% pluronic F-68, 5% glycerol) in a 100 MWCO centrifugal filter column (MilliporeSigma, Burlington, MA, USA). Samples were analyzed via silver stain to check purity and qPCR to quantify.

**Immune cell preparation, antibody staining, and flow cytometry.** Blood collected at the respective timepoints was transferred into EDTA capillary collection tubes (Microvette 600 K3E, Sarstedt, Nümbrecht, Germany). Red blood cells were lysed with 1 X lysis buffer (BD Pharm Lyse, BD Biosciences, San Jose, CA, USA) for 15 min in the dark at room temperature. Upon lysis and quenching with 10% (v/v) FBS DMEM media, the red blood cells were washed twice with 1% FBS-PBS solution. 2–4 × 10^6^ PBMCs were aliquoted per staining reaction and stained for 1 hour at 4 °C in the dark with the appropriate antibody mixtures. After being washed with 1% (v/v) FBS in PBS, cells were fixed in the dark for 20 min at 4°C with fixation buffer (1% (v/v) FBS, 4% (w/v) PFA in PBS). Flow cytometry was conducted using an LSRII Flow Cytometer (BD Biosciences) and data was analyzed using FlowJo software (TreeStar, Ashland, OR, USA). Flow cytometry fluorophore compensation for antibodies was conducted using AbC™ Anti-Mouse Bead Kit (Thermo Scientific, Waltham, MA). Counting beads were added to each sample prior flow-cytometry analysis (AccuCheck Counting Beads; Thermo Scientific, Invitrogen or Precision Count Beads; BD Biosciences. Chimerism of all humanized mouse models was assessed prior each experiment by quantifying the following human populations: Human CD45^+^, human CD45^+^ murine CD45^–^; T-cells, CD45^+^ CD3^+^; CD4^+^ T cells, CD45^+^ CD3^+^ CD4^+^; CD8^+^ T cells, CD45^+^ CD3^+^ CD8^+^; B cells, CD45^+^ CD3^–^ CD19^+^; Monocytes, CD45^+^ CD3^–^ CD19^–^ (CD56^–^) CD14^+^; Dendritic cells, CD45^+^ CD3^–^ CD19^–^ (CD56^–^) CD11c^+^; Natural Killer cells, CD45^+^ CD3^–^ (CD19^–^) CD56^+^. Human immune cell subsets were gated as followed: Human CD45^+^, human CD45^+^ murine CD45^–^; Natural Killer cells, CD45^+^ CD3^–^ (CD19^–^) CD56^+^; Natural Killer T cells and γδ T cells, CD45^+^ CD3^+^ (CD19^–^) CD56^+^; Plasmacytoid dendritic cells, CD45^+^ CD3^–^ CD19^–^ (CD56^–^) BDCA-2^+^ CD123^+^. For T-cell phenotyping, PBMC-derived CD8^+^ T cells were analyzed for their expression of CD38 and HLA-DR. Subpopulations were qualiﬁed as follow: naive CD8^+^ T-cells, CD8^+^ CD38^–^ HLA-DR^–^; activated CD8^+^ T cells, CD8^+^ CD38^+^ HLA-DR^+^. For platelet counts, human platelet, hCD41a^+^ mCD41^–^; murine platelet, hCD41a^–^ mCD41a^+^.

**SUPPLEMENTARY FIGURES**

**Supplementary Figure 1. Experimental workflow of the present study.** The 20 clinical isolates were obtained from the World Reference Center for Emerging Viruses and Arboviruses (WRCEVA). After reconstitution of the viruses by PBS, the individual viruses were subjected to amplification into the mammalian Huh7.5 cells. After collection of the supernatants, each viral RNA was extracted and used for determining sequence of whole virus genome. The remaining virus was stocked at –80°C. After sequencing of whole virus genome, each virus was used for molecular cloning, *in vitro*, and *in vivo* characterization. Illustration was created with BioRender.com.

**Supplementary Figure 2. Nucleotide alignment of the 20 clinical isolates of DENV.**

Alignment of the (A) 5′ UTR and (B) 3′ UTR of the 20 clinical isolates of DENV along with the reference strain of each of the 4 serotypes. Color indicates the respective nucleotide. Numbers starts from beginning of the individual UTR sequence. * indicates nucleotide identities. SLA: stem-loop A, SLB: stem-loop B, SL1: stem-loop 1, SL2: stem-loop 2, PK-DB1: pseudoknot dumbbell-like stem-loop 1, PK-DB2: pseudoknot dumbbell-like stem-loop 2, CS: conserved sequences, 3'DAR: 3' downstream of AUG region, sHP: short hairpin, 3'UAR: 3' upstream of AUG region, 3'SL: 3' stem-loop.

**Supplementary Figure 3. Experimental workflow for the generation of infectious DENV clones from clinical isolates by circular polymerase extension reaction (CPER). The seven synthetic DNA fragments covering the whole virus genome of the respective strains.** The fragments were subcloned into TOPO vectors and their sequences confirmed. Using these plasmids as templates the fragments were amplified with a high-fidelity DNA polymerase. The 7 purified PCR amplicons were mixed with the UTR linker, which contains a CMV promoter and a HDVr site at the 3' end and subjected to CPER reaction. The CMV immediate-early enhancer and promotor allows for constitutive expression in mammalian cells and initiates viral RNA transcription, while the HDVr site facilitates the authentic formation of the UTR termini. The CPER product was transfected into the Huh7.5 producer cells. The supernatants were harvested until CPE was observed**.** Illustration was created with BioRender.com.

**Supplementary Figure 4. Human hematopoietic reconstitution of the HIS-NFA2 mice used in this study prior to AAV-FLT3Lg treatment.**

Frequencies of each cell population is shown as a percentage of human CD45^+^ cells, with the exception of CD4^+^ and CD8^+^ T cells, which are displayed as a percentage of human CD3^+^ T cells. Medians are shown for each cell subset frequency as horizontal black line (*n* = 18).

**Supplementary Figure 5. AAV-mediated expression of hFLT3Lg results in expansion of human myeloid and natural killer cells in HIS-NFA2 mice.** (A) Human FLT3Lg concentration in the sera of non-engrafted NRG mice following injection of AAV-FLT3Lg (10^10^, 10^11^, or 10^12^ particles per mouse, *n* = 3 per group and dose). (B) Human FLT3Lg concentration in the sera of HIS-NFA2 mice following infection with 10^12^ AAV-FLT3Lg particles. Medians are shown as horizontal black lines (*n* = 3 per group). (C) Frequencies of human plasmacytoid dendritic cells (pDCs) and natural killer (NK) cells in HIS-NFA2 mice prior to and at day 10 post AAV-FLT3Lg injection. Medians are shown as horizontal black lines (*n* = 3 per group).

**Supplementary Figure 6. Comparison of the impact of inocula and routes of administration on DENV viremia in HIS-NFA2/hFLT3Lg mice.** DENV RNA copy numbers in HIS-NFA2/hFLT3Lg mice infected with DENV2 INS378816 strain. Three cohorts of mice were inoculated either i.v. with 7x10^5^ FFU of DENV2 (*n* = 5, cyan), s.c. with 7x10^5^ FFU of DENV2 (*n* = 3, red), i.v. with 7x10^4^ FFU of DENV2 (*n* = 5, blue), or s.c. with 7x10^4^ FFU of DENV2 (*n* = 5, white), Mice were bled at indicated timepoints post-infection, and viral RNA was extracted from plasma and quantified by RT-qPCR. The dashed line represents the threshold for viral RNA detection (1,500 RNA copies/ml). Each symbol represents one mouse.

**Supplementary** **Figure 7. Ratio of human CD45^+^ cells in HIS-NFA2/hFLT3Lg mice during DENV infection.**

Blood samples from the infected HIS-NFA2/hFLT3Lg mice were collected before (day 0) and after days 0 5, 10, 15, 20 post-infection. Samples were stained for hCD45^+^ and mCD45^+^ to evaluate the ratio of chimerism.

**Supplementary Figure 8. Murine platelets remain largely stable during DENV infection.**

Murine platelet counts were assessed longitudinally over the course of 20 days after DENV-infection. Mice from each of the 4 experimental cohorts were bled at the indicated timepoints. Samples were stained for mCD41a^+^ to assess the absolute cell count per microliter of blood. The numbers are normalized on the day of DENV- infection (baseline). Statistical significance was assessed using Friedman with Dunnett’s test and is indicated by asterisks (*, compared between baseline).

**SUPPLEMENTARY TABLES**

| **Virus ID** | **Strain Designation** | **Country of isolate** | **Year of isolation** | **DDBJ/ENA/GenBank accession number** |
| --- | --- | --- | --- | --- |
| DENV 1-1 | AUS H TI 9 | Australia | 1983 | OK605753 |
| DENV 1-2 | BE H 584526 | Brazil | 1997 | OK605754 |
| DENV 1-3 | C 0268-96 | Thailand | 1996 | OK605755 |
| DENV 1-4 | FSE 2133 | Ecuador | 2007 | OK605756 |
| DENV 1-5 | RIO H 36589 | Angola | 1988 | OL452067 |
| DENV 2-1 | DAK HD 76395 | Senegal | 1990 | OK605757 |
| DENV 2-2 | INS 378816 | Colombia | 1998 | OK605758 |
| DENV 2-3 | K 0006-94 | Thailand | 1994 | OK605759 |
| DENV 2-4 | PH H 023-88 | Philippines | 1988 | OK605760 |
| DENV 2-5 | SP 194589 | Brazil | 2000 | OK605761 |
| DENV 3-1 | FPA 0099 | Paraguay | 2007 | OK605762 |
| DENV 3-2 | JKT 85-934 | Indonesia | 1985 | OK605763 |
| DENV 3-3 | K 0077-94 | Thailand | 1994 | OK605764 |
| DENV 3-4 | OBT 1477 | Ecuador | 2001 | OK605765 |
| DENV 3-5 | S 162 | Somalia | 1993-1994 | OK605766 |
| DENV 4-1 | 1229 | Indonesia | 1976 | OK605767 |
| DENV 4-2 | BE H 403714 | Brazil | 1982 | OK605768 |
| DENV 4-3 | DAK H 38550 | Senegal | 1983 | OK605769 |
| DENV 4-4 | FSE 2098 | Ecuador | 2006 | OK605770 |
| DENV 4-5 | K 0036/94 | Thailand | 1994 | OK605771 |

**Supplementary Table 1.** **Information of the Dengue viruses used in this study.** The twenty clinical isolates used in this study were obtained from the World Reference Center for Emerging Viruses and Arboviruses (WRCEVA). The strain ID, country, and year of isolation, and GeneBank accession number are shown.

**Supplementary Table 2. Amino acid differences of viral polyprotein.**

The percentage of amino acid similarity compared with whole virus sequence is shown.

| **Primer ID** | **Orientation** | **Nucleotide sequence (5’−3’)** |
| --- | --- | --- |
| DENV-1 1F | Forward | GATCTCTGATGAACAACCAACG |
| DENV-1 1R | Reverse | CTTTTTATGGCCTCACGGACTATGGC |
| DENV-1 2F | Forward | CGTGTTCCACACAATGTGGC |
| DENV-1 2R | Reverse | GCTGTAGAGACAGCAGGATCTCTGGTC |
| DENV-2 1F | Forward | CAGATCTCTGATGAATAACCAACG |
| DENV-2 1R | Reverse | CTCCTGGGTGGAGGTCCATGATGGTC |
| DENV-2 2F | Forward | GACTTTTCTCCTGGAACGTCAGGATCTC |
| DENV-2 2R | Reverse | CATTTTCTGGCGTTCTGTGC |
| DENV-3 1F | Forward | GAAGCTTGCTTAACGTAGTGC |
| DENV-3 1R | Reverse | CACTCCATTGCCATACAGTC |
| DENV-3 2F | Forward | GTGCAGGTTATTGCCGTAGAG |
| DENV-3 2R | Reverse | GTTCTGTGCCTGGAATGATGC |
| DENV-4 1F | Forward | CACCTTTCAATATGCTGAAACGC |
| DENV-4 1R | Reverse | CTCGATGTCTTCTATTGGGCTG |
| DENV-4 2F | Forward | GAGCCAGATTATGAAGTGGATGAG |
| DENV-4 2R | Reverse | CGCTCTGTGCCTGGATTGATG |

**Supplementary Table 3.** **Primer sets utilized for PCR amplicons.** The serotype specific reverse primer was used to synthesize cDNA of reach virus RNA. The respective forward and reverse primer set was for generating PCR amplicon for the following NGS sequence.

| **Primer ID** | **Orientation** | **Nucleotide sequence (5’−3’)** |
| --- | --- | --- |
| DENV-1 circular F | Forward | TGGTGGTAAGGACTAGAG |
| DENV-1 circular R | Reverse | GAGAATCTCTTCGCCAACTG |
| DENV-2 circular F | Forward | GACCAGAGATCCTGCTGTC |
| DENV-2 circular R | Reverse | CTCTCGCGTTTCAGCATATTG |
| DENV-3 circular F | Forward | CATATTGACGCTGGGAGAGAC |
| DENV-3 circular R | Reverse | CTGTGATCCAGTTGACACACG |
| DENV-4 circular F | Forward | CAAACCGTGCTGCCTGTAG |
| DENV-4 circular R | Reverse | CACCATCCGTAAGGGTCCTTTC |

**Supplementary Table 4.** **Primer sets utilized for PCR of circular cDNAs.**

The serotype specific reverse primer was used to synthesize circular cDNA and the forward/reverse primer set for determination of 5' and 3' UTR sequence.

| **Fragment** | **Primer ID** | **Orientation** | **Nucleotide sequence (5’−3’)** |
| --- | --- | --- | --- |
| DENV-1 Fragment 1 | DENV-1 F1-F | Forward | AGTTGTTAGTCTACGTGGACC |
|  | DENV-1 F1-R | Reverse | GAACCAGCTTAGTTTCAAAG |
| DENV-1 Fragment 2 | DENV-1 F2-F | Forward | CTTTGAAACTAAGCTGGTTC |
|  | DENV-1 F2-R | Reverse | CATGCCTCCAGCTATTAGTG |
| DENV-1 Fragment 3 | DENV-1 F3-F | Forward | CACTAATAGCTGGAGGCATG |
|  | DENV-1 F3-R | Reverse | GCTGCACTCTTTTCTCTCTC |
| DENV-1 Fragment 4 | DENV-1 F4-F | Forward | GAGAGAGAAAAGAGTGCAGC |
|  | DENV-1 F4-R | Reverse | ACAAACCTATCACCACATATG |
| DENV-1 Fragment 5 | DENV-1 F5-F | Forward | CATATGTGGTGATAGGTTTGT |
|  | DENV-1 F5-R | Reverse | CTCCATGTTTTCTTTGCAT |
| DENV-1 Fragment 6 | DENV-1 F6-F | Forward | ATGCAAAGAAAACATGGAG |
|  | DENV-1 F6-R | Reverse | CTATCCAAACCCTATTCCAC |
| DENV-1 Fragment 7 | DENV-1 F7-F | Forward | GTGGAATAGGGTTTGGATAG |
|  | DENV-1 F7-R | Reverse | AGAACCTGTTGATTCAACAGC |
| DENV-1 Fragment 8 | DENV-1 F8-F | Forward | TGCTGTTGAATCAACAGGTTCTGGGTCGGCATGGCATCTCCAC |
|  | DENV-1 F8-R | Reverse | CGGTCCACGTAGACTAACAACTCGGTTCACTAAACGAGCTCTGC |
| DENV-2 Fragment 1 | DENV-2 F1-F | Forward | AGTTGTTAGTCTACGTGGAC |
|  | DENV-2 F1-R | Reverse | GTTCTGCTTCTATGTTGACTG |
| DENV-2 Fragment 2 | DENV-2 F2-F | Forward | CAGTCAACATAGAAGCAGAAC |
|  | DENV-2 F2-R | Reverse | CTCAAGAGTAGTCCAGCTG |
| DENV-2 Fragment 3 | DENV-2 F3-F | Forward | CAGCTGGACTACTCTTGAG |
|  | DENV-2 F3-R | Reverse | CTCTTCTTTGTGCTGCACTA |
| DENV-2 Fragment 4 | DENV-2 F4-F | Forward | TAGTGCAGCACAAAGAAGAG |
|  | DENV-2 F4-R | Reverse | TTGAAGCTGCTATCCAGTGT |
| DENV-2 Fragment 5 | DENV-2 F5-F | Forward | ACACTGGATAGCAGCTTCAA |
|  | DENV-2 F5-R | Reverse | GCTCCTCCATATTTCCTTTG |
| DENV-2 Fragment 6 | DENV-2 F6-F | Forward | CAAAGGAAATATGGAGGAGC |
|  | DENV-2 F6-R | Reverse | AGCCGCACCATTGGTCTTCT |
| DENV-2 Fragment 7 | DENV-2 F7-F | Forward | AGAAGACCAATGGTGCGGCT |
|  | DENV-2 F7-R | Reverse | AGAACCTGTTGATTCAACAG |
| DENV-2 Fragment 8 | DENV-2 F8-F | Forward | CTGTTGAATCAACAGGTTCTGGGTCGGCATGGCATCTCCACCTC |
|  | DENV-2 F8-R | Reverse | GTCCACGTAGACTAACAACTCGGTTCACTAAACGAGCTCTGC |
| DENV-2 sylvatic Fragment 1 | DENV-2 sylvatic F1-F | Forward | AGTTGTTAGTCTACGTGGACCG |
|  | DENV-2 sylvatic F1-R | Reverse | CATTCTTTTAGCTCCTCTCATTG |
| DENV-2 sylvatic Fragment 2 | DENV-2 sylvatic F2-F | Forward | CAATGAGAGGAGCTAAAAGAATG |
|  | DENV-2 sylvatic F2-R | Reverse | GCTTCATTAAGAGGCCAACTTC |
| DENV-2 sylvatic Fragment 3 | DENV-2 sylvatic F3-F | Forward | GAAGTTGGCCTCTTAATGAAGC |
|  | DENV-2 sylvatic F3-R | Reverse | GAACATGCTGGGGATGATAC |
| DENV-2 sylvatic Fragment 4 | DENV-2 sylvatic F4-F | Forward | GTATCATCCCCAGCATGTTC |
|  | DENV-2 sylvatic F4-R | Reverse | GCAGGTCGGAGGTCTATGTC |
| DENV-2 sylvatic Fragment 5 | DENV-2 sylvatic F5-F | Forward | GACATAGACCTCCGACCTGC |
|  | DENV-2 sylvatic F5-R | Reverse | CTTGTGCCTCATTGTGAATC |
| DENV-2 sylvatic Fragment 6 | DENV-2 sylvatic F6-F | Forward | GATTCACAATGAGGCACAAG |
|  | DENV-2 sylvatic F6-R | Reverse | GTATGTCTTCCCATGATTCCAC |
| DENV-2 sylvatic Fragment 7 | DENV-2 sylvatic F7-F | Forward | GTGGAATCATGGGAAGACATAC |
|  | DENV-2 sylvatic F7-R | Reverse | AGAACCTGTTGATTCAACAG |
| DENV-2 sylvatic Fragment 8 | DENV-2 sylvatic F8-F | Forward | GTGCTGTTGAATCAACAGGTTCGGGTCGGCATGGCATCTCCAC |
|  | DENV-2 sylvatic F8-R | Reverse | CGGTCCACGTAGACTAACAACTCGGTTCACTAAACGAGCTCTG |
| DENV-3 Fragment 1 | DENV-3 F1-F | Forward | AGTTGTTAGTCTACGTGGAC |
|  | DENV-3 F1-R | Reverse | CACCACCCACTGATCCAAAG |
| DENV-3 Fragment 2 | DENV-3 F2-F | Forward | CTTTGGATCAGTGGGTGGTG |
|  | DENV-3 F2-R | Reverse | CATTGTTCCATCATCATCAAC |
| DENV-3 Fragment 3 | DENV-3 F3-F | Forward | GTTGATGATGATGGAACAATG |
|  | DENV-3 F3-R | Reverse | CAACTCTCCCTCTCCTTTGCGC |
| DENV-3 Fragment 4 | DENV-3 F4-F | Forward | GCGCAAAGGAGAGGGAGAGTTG |
|  | DENV-3 F4-R | Reverse | GACATATGCGAGTTGGTTGTC |
| DENV-3 Fragment 5 | DENV-3 F5-F | Forward | GACAACCAACTCGCATATGTC |
|  | DENV-3 F5-R | Reverse | GTTGGGCTTGGTGAAGATTC |
| DENV-3 Fragment 6 | DENV-3 F6-F | Forward | GAATCTTCACCAAGCCCAAC |
|  | DENV-3 F6-R | Reverse | GTTCCAGACAGTAAGCATGTC |
| DENV-3 Fragment 7 | DENV-3 F7-F | Forward | GACATGCTTACTGTCTGGAAC |
|  | DENV-3 F7-R | Reverse | AGAACCTGTTGATTCAACAG |
| DENV-3 Fragment 8 | DENV-3 F8-F | Forward | CTGTTGAATCAACAGGTTCTGGGTCGGCATGGCATCTCCACCTC |
|  | DENV-3 F8-R | Reverse | GTCCACGTAGACTAACAACTCGGTTCACTAAACGAGCTCTGC |
| DENV-4 Fragment 1 | DENV-4 F1-F | Forward | AGTTGTTAGTCTGTGTGGACCGACAAG |
|  | DENV-4 F1-R | Reverse | GCTGTGTTTCTGCCATCTC |
| DENV-4 Fragment 2 | DENV-4 F2-F | Forward | GAGATGGCAGAAACACAGC |
|  | DENV-4 F2-R | Reverse | CATCTTCATCCTGCTTCACTTC |
| DENV-4 Fragment 3 | DENV-4 F3-F | Forward | GAAGTGAAGCAGGATGAAGATG |
|  | DENV-4 F3-R | Reverse | ACTCTCCATCAATGGCTTG |
| DENV-4 Fragment 4 | DENV-4 F4-F | Forward | CAAGCCATTGATGGAGAGT |
|  | DENV-4 F4-R | Reverse | GACGTAGATCAATTGATTGTC |
| DENV-4 Fragment 5 | DENV-4 F5-F | Forward | GACAATCAATTGATCTACGTC |
|  | DENV-4 F5-R | Reverse | CTATGACTGTTGGCATGTAG |
| DENV-4 Fragment 6 | DENV-4 F6-F | Forward | CTACATGCCAACAGTCATAG |
|  | DENV-4 F6-R | Reverse | GCATATCTTCAGTGGTCATC |
| DENV-4 Fragment 7 | DENV-4 F7-F | Forward | GATGACCACTGAAGATATGC |
|  | DENV-4 F7-R | Reverse | AGAACCTGTTGGATCAACAAC |
| DENV-4 Fragment 8 | DENV-4 F8-F | Forward | TGTTGTTGATCCAACAGGTTCTGGGTCGGCATGGCATCTCCACCTC |
|  | DENV-4 F8-R | Reverse | CGGTCCACACAGACTAACAACTCGGTTCACTAAACGAGCTCTGC |

**Supplementary Table 5.** **Primer sets utilized for CPER.**

The serotype specific primer set was used to PCR amplicon for the CPER reaction. Note: the DENV2-1 DAK HD 76395 is a sylvatic strain.

**Supplementary References**

1. Wick RR, Judd LM, Gorrie CL, et al. Unicycler: Resolving bacterial genome assemblies from short and long sequencing reads. PLoS Comput Biol. 2017 Jun;13(6):e1005595.

2. Hadfield J, Megill C, Bell SM, et al. Nextstrain: real-time tracking of pathogen evolution. Bioinformatics. 2018 Dec 1;34(23):4121-4123.

3. Edgar RC. MUSCLE: multiple sequence alignment with high accuracy and high throughput. Nucleic Acids Res. 2004;32(5):1792-7.

4. Larkin MA, Blackshields G, Brown NP, et al. Clustal W and Clustal X version 2.0. Bioinformatics. 2007 Nov 1;23(21):2947-8.
